# circ_0085296 inhibits the biological functions of trophoblast cells to promote the progression of preeclampsia via the miR-942-5p/THBS2 network

**DOI:** 10.1515/med-2022-0427

**Published:** 2022-03-21

**Authors:** Jiyi Liu, Yan Yang, Wenlan Liu, Ruilun Lan

**Affiliations:** Department of Obstetric, Jiangjin Maternal and Child Health Hospital, Jiangjin District, Chongqing, 402260, China; Department of Obstetric, Jiangjin Maternal and Child Health Hospital, 192 Jiangzhou Dadao, Jiangjin District, Chongqing, 402260, China

**Keywords:** preeclampsia, trophoblast cells, circ_0085296, miR-942-5p, THBS2

## Abstract

Insufficient invasion of trophoblast cells is one of the important causes of preeclampsia (PE). Circular RNA (circRNA) has been proven to regulate the biological functions of trophoblast cells and mediate the progression of PE. The expression of circ_0085296, microRNA (miR)-942-5p, and thrombospondin 2 (THBS2) was detected by quantitative real-time PCR. In addition, the interaction between miR-942-5p and circ_0085296 or THBS2 was confirmed by dual-luciferase reporter assay and RIP assay. Our data showed that circ_0085296 was upregulated in the placental tissues of PE patients. Silenced circ_0085296 could enhance the proliferation, migration, invasion, and angiogenesis of HTR-8/SVneo cells. Besides, circ_0085296 was found to act as miR-942-5p sponge. Function analysis results suggested that miR-942-5p inhibitor reversed the positive regulation of circ_0085296 knockdown on the biological functions of HTR-8/SVneo cells. Moreover, THBS2 was a target of miR-942-5p, and its overexpression also reversed the promotion effect of miR-942-5p on the proliferation, migration, invasion, and angiogenesis of HTR-8/SVneo cells. Also, circ_0085296 was discovered to positively regulate THBS2 by sponging miR-942-5p. To sum up, our results revealed that circ_0085296 could inhibit trophoblast cells proliferation, migration, invasion, and angiogenesis by regulating miR-942-5p/THBS2, confirming that circ_0085296 might be a potential therapeutic target for PE.

## 1 Introduction

Preeclampsia (PE) is a leading cause of maternal mortality and is characterized by high blood pressure after 20 weeks of gestation [1,2]. The incidence of PE has shown an upward trend year by year. How to effectively reduce the mortality risk of pregnant women and fetuses to improve the survival rate has become an urgent matter in contemporary obstetrics and gynecology medical research [3,4]. At present, it is believed that the possible cause of PE is that the insufficiency of trophoblast cell invasion in the early pregnancy causes the uterine spiral artery reconstruction disorder [5,6]. Therefore, elucidating the molecular mechanisms affecting the biological functions of trophoblast cells is expected to provide a theoretical basis for improving PE progression.

Circular RNA (circRNA) is a non-coding RNA with regulatory functions, which is abundantly present in the eukaryotic transcriptome [7,8]. In recent years, many studies have found that circRNA located in the cytoplasm can compete with mRNA for the targeted binding site of microRNA (miRNA), thereby regulating mRNA expression, which is called the competing endogenous RNA (ceRNA) mechanism [9,10]. Today, circRNAs have been shown to play a vital role in the human diseases, including PE [11,12]. For example, circTNRC18 suppressed the migration of trophoblast cells via miR-762/Grhl2 axis, confirming that circTNRC18 might promote PE progression [13]. On the contrary, circPAPPA regulated miR-384/STAT3 axis to inhibit PE progression, knockdown of which inhibited trophoblast cell proliferation and invasion [14]. Hence, circRNA is a key regulator of PE progression.

circ_0085296 is located at chr8: 104922361–105026843 with 1728 bp, and is derived from RIMS2 gene. A recent study had shown that circ_0085296 was significantly overexpressed in PE patients and could regulate trophoblast cell proliferation, migration, and invasion [15]. Therefore, more evidence is needed to further verify the key role of circ_0085296 in PE progression and provide a new theoretical basis for circ_0085296 to become a potential target of PE treatment. Our study aims to reveal the effect and underlying molecular mechanism of circ_0085296 in the biological functions of trophoblast cells to confirm its role in PE progression.

## 2 Materials and methods

### 2.1 Samples

After cesarean section, the placental tissues of 35 PE patients and 35 normal pregnant women were collected from Jiangjin Maternal and Child Health Hospital. The placental tissues were stored at −80°C until used. For our research, each patient and normal pregnant woman signed written informed consent. Our study was approved by the Ethics Committee of Jiangjin Maternal and Child Health Hospital.

### 2.2 Cell culture

Human chorionic trophoblast cells (HTR-8/SVneo; ATCC, Manassas, VA, USA) were cultured in RPMI-1640 medium (Gibco, Grand Island, NY, USA) containing 10% FBS (Gibco) and 1% penicillin–streptomycin (Sangon, Shanghai, China) at 37°C with 5% CO_2_.

### 2.3 Cell transfection

The oligonucleotides and vectors were synthesized by RiboBio (Guangzhou, China), including circ_0085296 siRNA and overexpression vector (si-circ_0085296#1/#2 and circ_0085296), miR-942-5p mimic and inhibitor (miR-942-5p and anti-miR-942-5p), and pcDNA thrombospondin 2 (THBS2) overexpression vector and their negative controls. Lipofectamine 3000 (Invitrogen, Carlsbad, CA, USA) was used to perform cell transfection.

### 2.4 Quantitative real-time PCR (qRT-PCR)

According to the instructions of TRIzol reagent (Invitrogen), total RNA was isolated. cDNA was synthesized using PrimeScript RT Master Mix (Takara, Dalian, China), and PCR operation was performed using Power SYBR Green (Takara) and specific primers. Gene expression was normalized by GAPDH or U6 and calculated by 2^−ΔΔCt^ method. The specific primer sequences are exhibited in Table 1.

**Table 1:** Primer sequences for qRT-PCR

Genes	Primers for PCR (5′-3′)
circ_0085296	F: TCCAGACAAGCCCATCAAGT
	R: TGCCAAGACGTATGATCCAA
RIMS2	F: ACAGTATGCTACTTCGGATACCG
	R: CGTGACTGGTACTCTTCCTCTC
miR-942-5p	F: GCCGAGTCTTCTCTGTTTTGGC
	R: CTCAACTGGTGTCGTGGA
THBS2	F: GACACGCTGGATCTCACCTAC
	R: GAAGCTGTCTATGAGGTCGCA
U6	F: CGCTTCGGCAGCACATATACTA
	R: CGCTTCACGAATTTGCGTGTCA
GAPDH	F: CAATGACCCCTTCATTGACC
	R: TGGAAGATGGTGATGGGATT

### 2.5 Identification of circRNA

In RNase R assay, the extracted RNA was hatched with RNase R (Geneseed, Guangzhou, China) and then RNA was used for performing qRT-PCR to detect circ_0085296 and linear RNA RIMS2 expression.

In Actinomycin D (ActD) assay, HTR-8/SVneo cells were treated with ActD (Beijing Leagene Biotech Co., Ltd, Beijing, China) for 0, 4, 8, 12, and 24 h. After that, the RNA was extracted to determine circ_0085296 and linear RNA RIMS2 expression using qRT-PCR.

### 2.6 Subcellular localization assay

Based on the instructions of PARIS Kit (Invitrogen), the cytoplasm and nuclear RNAs were isolated. Then, qRT-PCR was used to examine circ_0085296, GAPDH (cytoplasm control) and U6 (nuclear control) expression.

### 2.7 Cell proliferation assay

In cell counting kit 8 (CCK8) assay, HTR8/SVneo cells were seeded into 96-well plates (4 × 10^3^ cells/well) and incubated overnight at 37°C. At the indicated time points, CCK8 solution (Dojindo, Kumamoto, Japan) was added into cells for 4 h. After that, optical density (OD) value was analyzed at 450 nm to evaluate cell viability.

In colony formation assay, HTR8/SVneo cells were seeded in 6-well-plates (250 cells/well). 2 weeks later, the colonies were stained by crystal violet and then counted under microscope.

### 2.8 Cell migration and invasion assays

Transwell assay was performed to analyze cell migration and invasion. 24-well Transwell chambers (Corning Inc., Corning, NY, USA) were used to measure cell migration, which pre-coated with a Matrigel (Corning Inc.) were used to examine cell invasion. Transfected HTR-8/SVneo cells re-suspended with serum-free medium were seeded into the upper chambers, and then the culture plate was placed at 37°C for 24 h under the condition of serum medium filled in the lower chamber. After that, the cells were stained with crystal violet. Under the microscope (100×), 3 fields of view were randomly selected to count the number of migrated and invaded cells.

### 2.9 Cell angiogenesis assay

Tube formation assay was performed to assess cell angiogenesis ability. After transfection for 48 h, the cell medium of HTR-8/SVneo cells was obtained. After centrifuged, the medium supernatant was mixed with the fresh serum RPMI-1640 medium in a ratio of 1:1 to prepare the conditioned medium growth. HUVEC cells suspended with the conditioned medium growth were seeded into the 96-well plates pre-coated with growth factor reduced-Matrigel (Corning Inc.). After incubation for 24 h, the tube formation was observed under a microscope and the tube formation rate was counted by ImageJ software.

### 2.10 Western blot (WB) analysis

Total protein was collected by RIPA Lysis Buffer (Beyotime, Shanghai, China). After quantifying, the protein was isolated by SDS-PAGE gel and transferred onto PVDF membranes (Invitrogen). The membrane was hatched with non-fat milk and treated with primary antibodies (Abcam, Cambridge, MA, USA), including anti-PCNA (ab18197, 1:1,000), anti-Ki67 (1:1,000, ab16667), anti-MMP9 (1:1,000, ab38898), anti-MMP2 (ab97779, 1:1,000), anti-VEGFA (ab46154, 1:1,000), anti-THBS2 (ab84469, 1:1,000), and anti-GAPDH (ab9485, 1:2,500). Finally, the membrane was incubated with Goat Anti-Rabbit IgG (ab205718, 1:50,000) and then the protein signals were detected by Immobilon Western Chemilum HRP Substrate (Millipore, Billerica, MA, USA).

### 2.11 Dual-luciferase reporter assay

According to the bioinformatics analysis software (circInteractome and targetScan), we predicted the binding sites between miR-942-5p and circ_0085296 or THBS2 3′-UTR. The wild-type and mutate-type sequences of circ_0085296 or THBS2 3′-UTR were sub-cloned into the pmirGLO reporter vector to build the circ_0085296^WT/MUT^ or THBS2 3′-UTR^WT/MUT^ reporter vectors. The above reporter vectors and miR-942-5p mimic or miR-NC were co-transfected into 293T cells. 48 h later, the luciferase activity was monitored using Dual-Luciferase Reporter Gene Assay Kit (Beyotime).

### 2.12 RIP assay

Based on the instructions of Magna RIP Kit (Millipore), the lysates of HTR-8/SVneo cells were incubated with magnetic beads coupled with Anti-Ago2 or Anti-IgG. The enrichments of circ_0085296, miR-942-5p, and THBS2 were measured by qRT-PCR.

### 2.13 Statistical analysis

Data were expressed as mean values ± SD from three independent experiments. Differences between groups were analyzed by Student’s *t*-test or one-way ANOVA followed by Tukey’s *post-hoc* test. Statistical analysis was performed using Graphpad Prism 7.0 software. *P* < 0.05 was regarded as statistically significant.

## 3 Results

### 3.1 circ_0085296 expression was increased in PE patients

**Figure 1:**
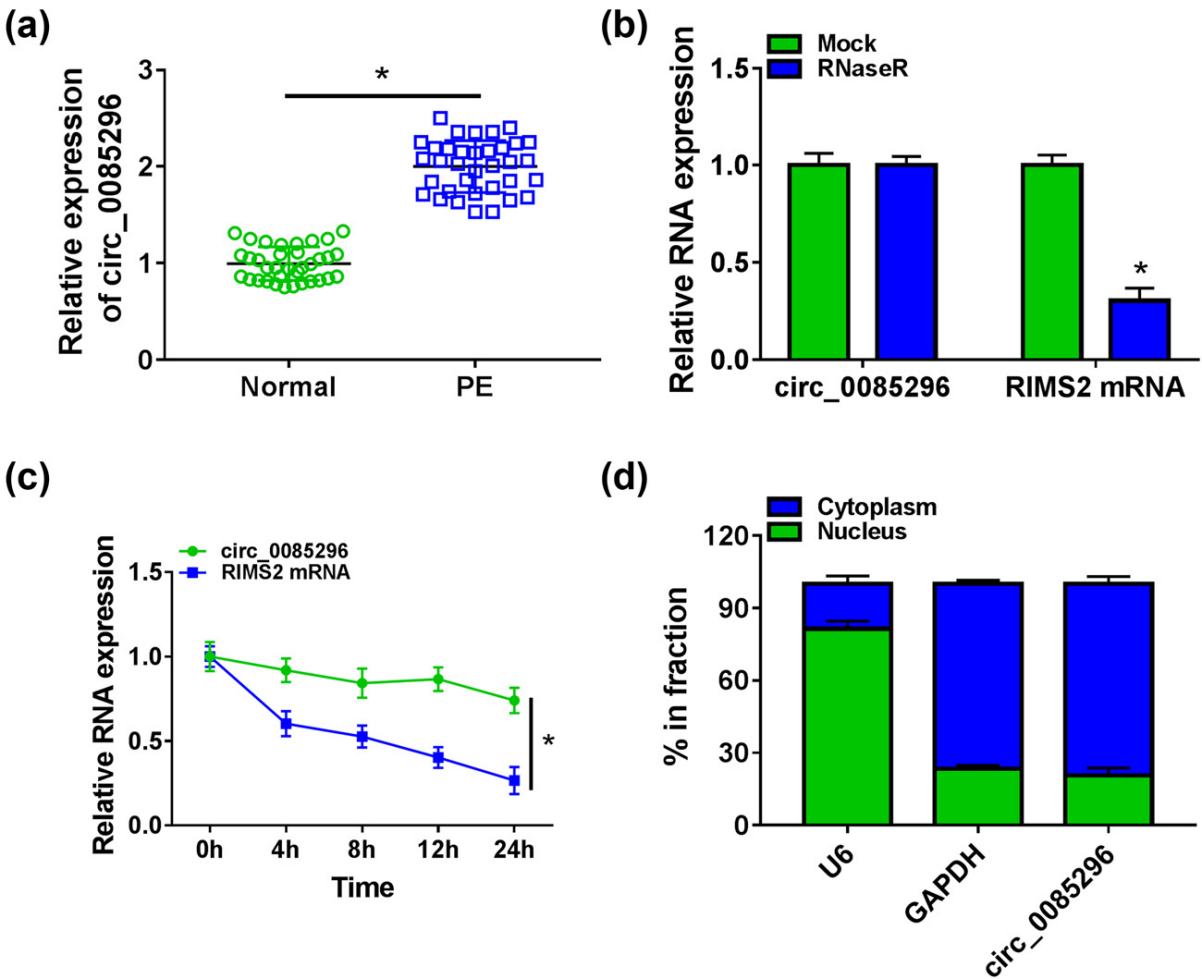
circ_0085296 expression was increased in PE patients. (a) The expression of circ_0085296 in the placental tissues of PE patients and normal pregnant women was measured by qRT-PCR. RNase R assay (b) and ActD assay (c) were performed to evaluate the circular characteristics of circ_0085296. (d) Subcellular localization assay was performed to assess the distribution of circ_0085296 in the cytoplasm and nucleus of HTR8/SVneo cells. *P < 0.05.

In the placental tissues of PE patients and normal pregnant women, we measured circ_0085296 expression and found that circ_0085296 was highly expressed in PE patients (Figure 1a). To confirm the circular characteristics of circ_0085296, we conducted RNase R assay and ActD assay. Compared to linear RNA RIMS2, circ_0085296 could resist the digestion of RNase and its expression was not affected under ActD treatment, suggesting that circ_0085296 had a stable circular structure. (Figure 1b and c). Moreover, the subcellular localization assay results also indicated that circ_0085296 was mainly distributed in the cytoplasm of HTR8/SVneo cells (Figure 1d). The above results confirmed that circ_0085296 was a highly expressed circRNA in PE patients, which might be mainly involved in post-transcriptional regulation.

### 3.2 Knockdown of circ_0085296 promoted HTR8/SVneo cells’ proliferation, migration, invasion, and angiogenesis

**Figure 2:**
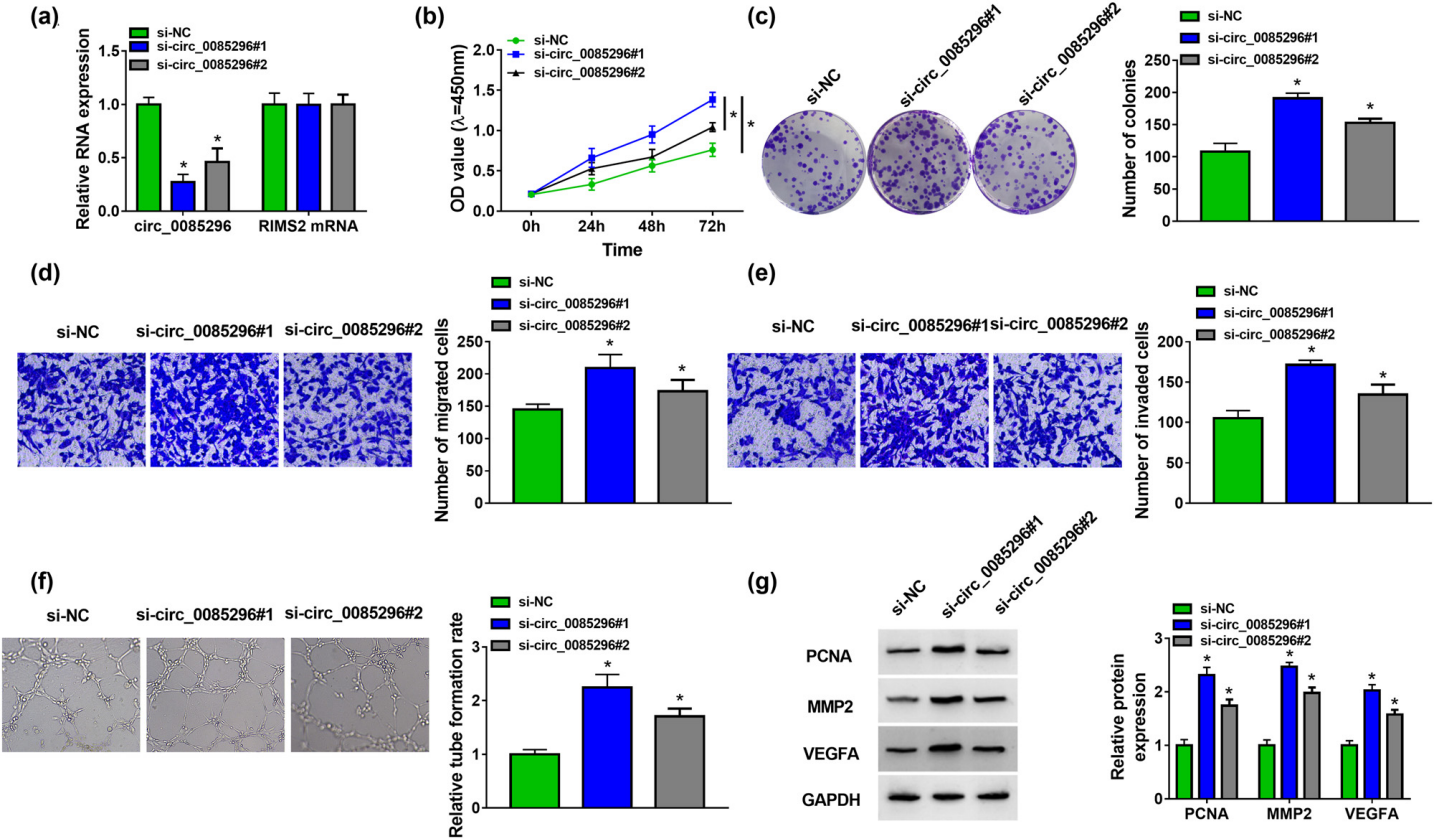
Effects of circ_0085296 knockdown on the biological functions of HTR8/SVneo cells. HTR-8/SVneo cells were transfected with si-NC or si-circ_0085296#1/#2. (a) The expression of circ_0085296 and RIMS2 was detected by qRT-PCR. CCK8 assay (b), colony formation assay (c), Transwell assay (d and e), and tube formation assay (f) were performed to measure cell proliferation, migration, invasion, and angiogenesis, respectively. (g) The protein levels of PCNA, MMP2, and VEGFA were tested by WB analysis. *P < 0.05.

**Figure A1:**
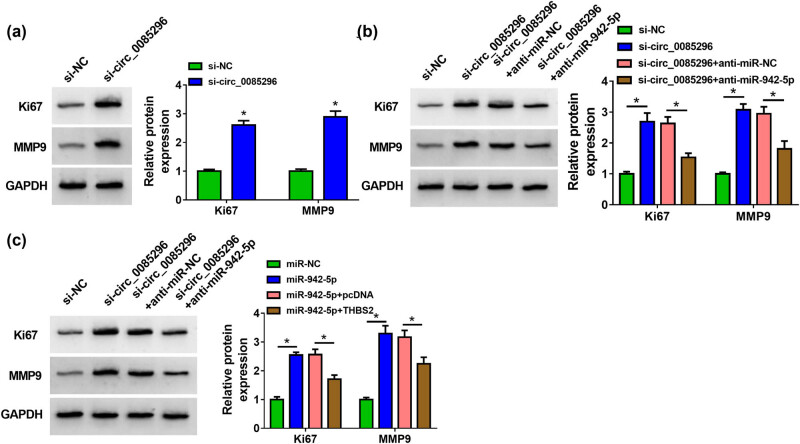
Effects of circ_0085296/miR-942-5p/THBS2 on Ki67 and MMP9 protein expression. (a) The Ki67 and MMP9 protein expression in HTR-8/SVneo cells transfected with si-NC or si-circ_0085296 was measured by WB analysis. (b) The Ki67 and MMP9 protein expression was determined by WB analysis in HTR-8/SVneo cells co-transfected with si-circ_0085296 and anti-miR-942-5p. (c) WB analysis was used to detect Ki67 and MMP9 proteins’ expression in HTR-8/SVneo cells co-transfected with miR-942-5p mimic and THBS2 overexpression vector. *P < 0.05.

To confirm the role of circ_0085296 in PE progression, we silenced circ_0085296 expression using si-circ_0085296#1 and si-circ_0085296#2. Our results showed that circ_0085296 knockdown only reduced the expression of circ_0085296, but had no effect on the expression of its linear RNA RIMS2 (Figure 2a). The results of CCK8 assay and colony formation assay revealed that circ_0085296 knockdown could promote the viability and the number of colonies in HTR8/SVneo cells (Figure 2b and c), suggesting that circ_0085296 might inhibit HTR8/SVneo cells’ proliferation. Transwell assay results showed that the number of migrated and invaded HTR8/SVneo cells were also enhanced by circ_0085296 knockdown (Figure 2d and e). Furthermore, tube formation assay was used to assess the angiogenesis ability of cells, and the results indicated that silenced circ_0085296 markedly promoted the tube formation rate of HTR8/SVneo cells (Figure 2f). In addition, we also measured the protein expression of proliferation markers (PCNA and Ki67), migration and invasion markers (MMP2 and MMP9), as well as angiogenesis marker (VEGFA), and found that circ_0085296 knockdown increased the protein expression of PCNA, Ki67, MMP9, MMP2, and VEGFA (Figure 2g and Figure A1a). These results showed that circ_0085296 could inhibit the proliferation, migration, invasion, and angiogenesis of HTR8/SVneo cells. In the following experiments, si-circ_0085296#1 was selected and simplified to si-circ_0085296.

### 3.3 circ_0085296 could serve as a sponge of miR-942-5p

**Figure 3:**
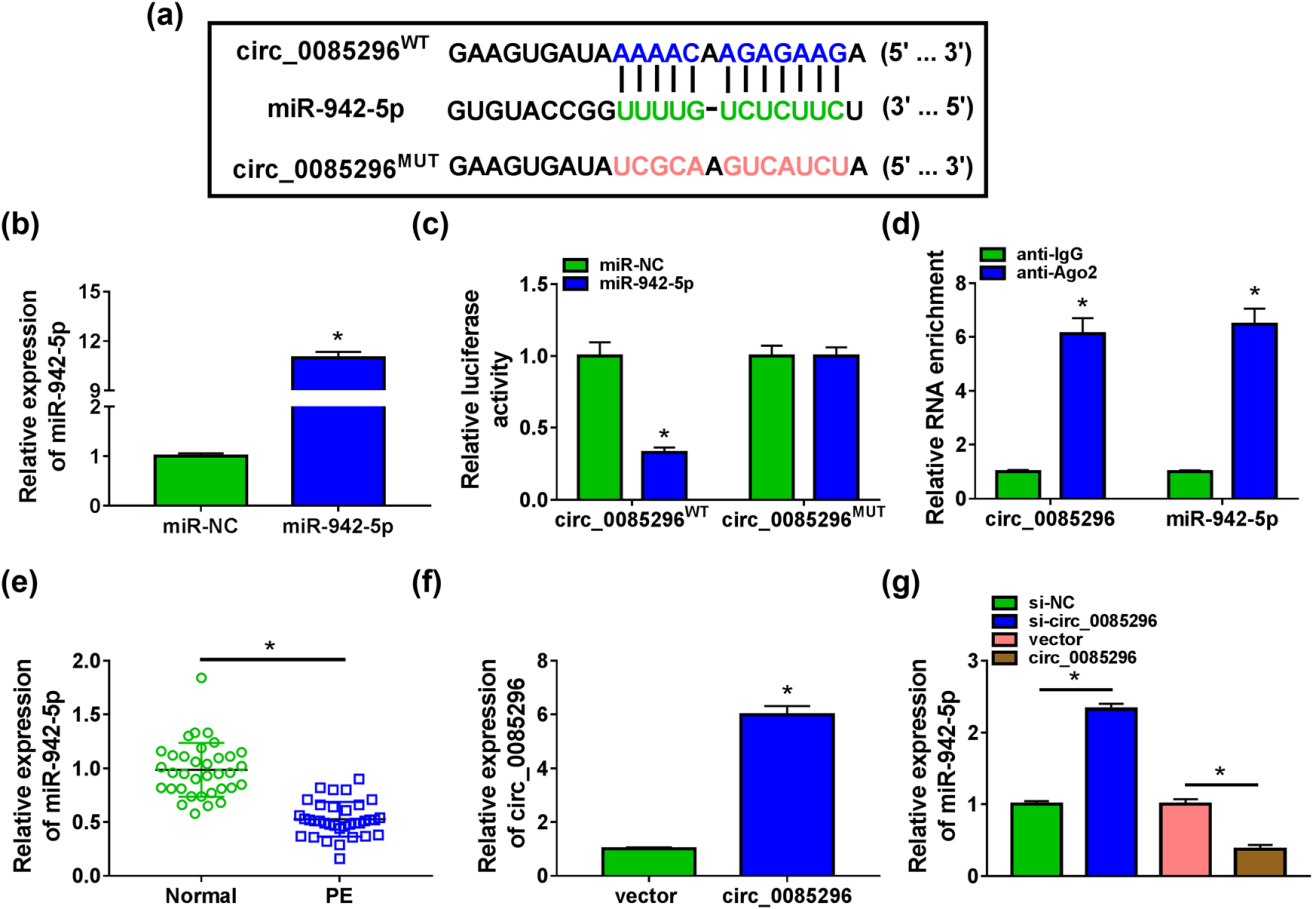
circ_0085296 sponged miR-942-5p. (a) The sequences of circ_0085296^WT/MUT^ vectors are shown. (b) The miR-942-5p expression was measured by qRT-PCR to assess the transfection efficiency of miR-942-5p mimic. Dual-luciferase reporter assay (c) and RIP assay (d) were performed to confirm the interaction between circ_0085296 and miR-942-5p. (e) The expression of miR-942-5p was examined by qRT-PCR in the placental tissues of PE patients and normal pregnant women. (f) The circ_0085296 expression was measured by qRT-PCR to evaluate the transfection efficiency of circ_0085296 overexpression vector. (g) QRT-PCR was used to detect miR-942-5p expression in HTR-8/SVneo cells transfected with si-circ_0085296 or circ_0085296 overexpression vector. *P < 0.05.

To perfect the mechanism of circ_0085296 regulated PE progression, we used the circInteractome software to predict the targeted miRNA for circ_0085296. Our data suggested that miR-942-5p had binding sites with circ_0085296 (Figure 3a). After transfecting HTR8/SVneo cells with miR-942-5p mimic, we found that miR-942-5p expression was markedly enhanced (Figure 3b). Then, miR-942-5p mimic and the circ_0085296^WT/MUT^ vectors were co-transfected into 293T cells to perform dual-luciferase reporter assay. The results showed that miR-942-5p mimic could reduce the luciferase activity of circ_0085296^WT^ vector without affecting that of the circ_0085296^MUT^ vector (Figure 3c). In addition, circ_0085296 and miR-942-5p could be enriched in anti-Ago2 (Figure 3d). These data verified the interaction between circ_0085296 and miR-942-5p. In the placental tissues of PE patients, we found that miR-942-5p was lowly expressed compared with that in normal pregnant women (Figure 3e). Then, we constructed circ_0085296 overexpression vector and confirmed that it could significantly elevate circ_0085296 expression (Figure 3f). By detecting miR-942-5p expression, we discovered that circ_0085296 knockdown could promote miR-942-5p expression in HTR8/SVneo cells, while its overexpression had an opposite effect (Figure 3g). The above results showed that circ_0085296 had a negative regulation on miR-942-5p expression.

### 3.4 MiR-942-5p inhibitor reversed the regulation of circ_0085296 knockdown on the biological functions of HTR8/SVneo cells

**Figure 4:**
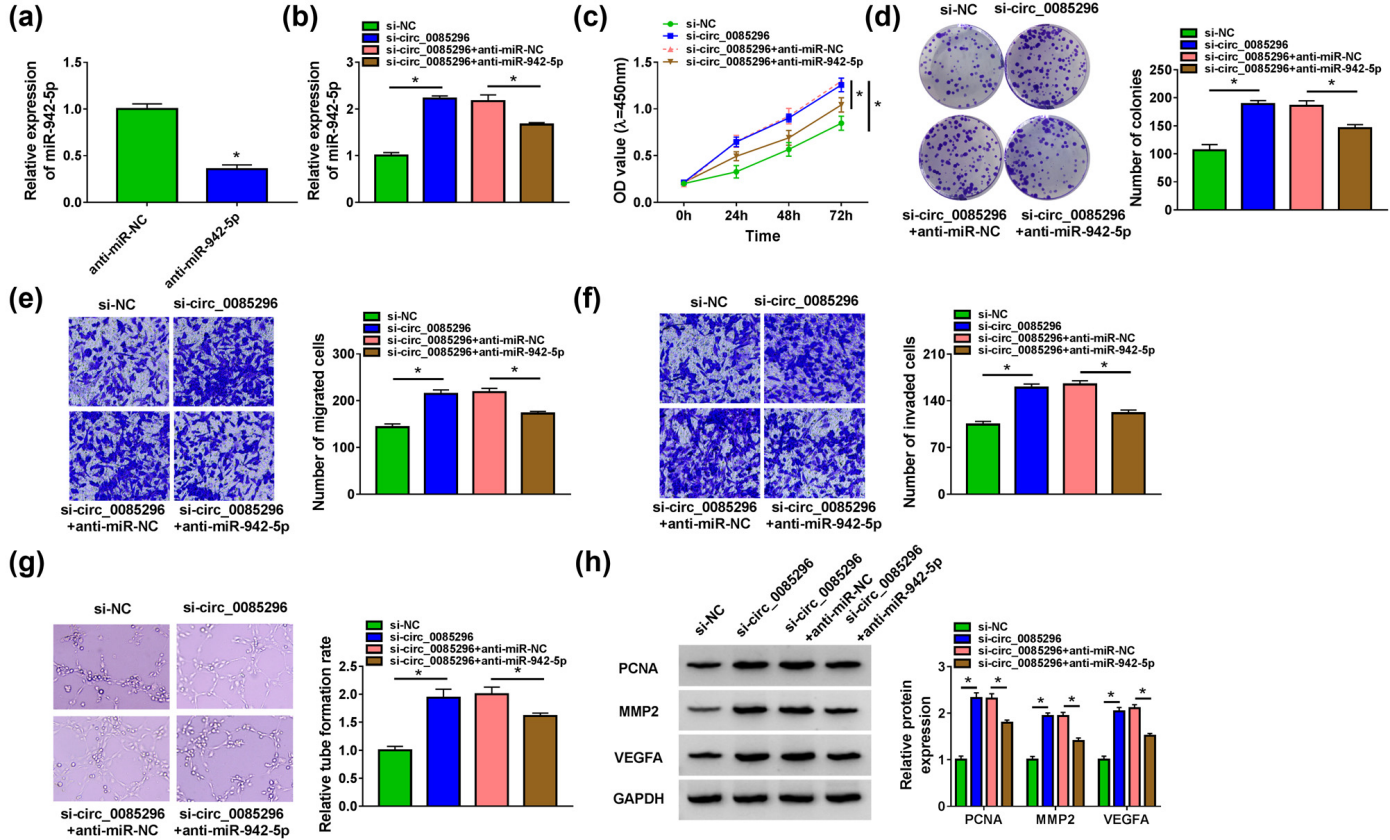
Effects of si-circ_0085296 and anti-miR-942-5p on the biological functions of HTR8/SVneo cells. (a) The miR-942-5p expression was detected by qRT-PCR to assess the transfection efficiency of anti-miR-942-5p. (b–h) HTR-8/SVneo cells were transfected with si-NC, si-circ_0085296, si-circ_0085296 + anti-miR-NC, or si-circ_0085296 + anti-miR-942-5p. (b) The expression of miR-942-5p was measured by qRT-PCR. Cell proliferation, migration, invasion, and angiogenesis were determined by CCK8 assay (c), colony formation assay (d), Transwell assay (e and f), and tube formation assay (g), respectively. (h) WB analysis was performed to examine the protein levels of PCNA, MMP2, and VEGFA. *P < 0.05.

To explore whether miR-942-5p participated in the regulation of circ_0085296 on PE progression, we performed the rescue experiments. After confirming that anti-miR-942-5p could decrease miR-942-5p (Figure 4a), we co-transfected si-circ_0085296 and anti-miR-942-5p into HTR8/SVneo cells. The increasing effect of si-circ_0085296 on miR-942-5p expression could be inhibited by anti-miR-942-5p (Figure 4b). Functional analysis results indicated that miR-942-5p inhibitor could reverse the promotion effect of circ_0085296 knockdown on the viability, the number of colonies, and the number of migrated and invaded cells (Figure 4c–f). Besides, the enhancing effect of circ_0085296 on the tube formation rate of HTR8/SVneo cells could also be partially abolished by miR-942-5p inhibitor (Figure 4g). In addition, the addition of anti-miR-942-5p also decreased the protein levels of PCNA, Ki67, MMP9, MMP2, and VEGFA increased by si-circ_0085296 in HTR8/SVneo cells (Figure 4h and Figure A1b). Therefore, our data showed that circ_0085296 promoted PE progression by sponging miR-942-5p.

### 3.5 MiR-942-5p could target THBS2

**Figure 5:**
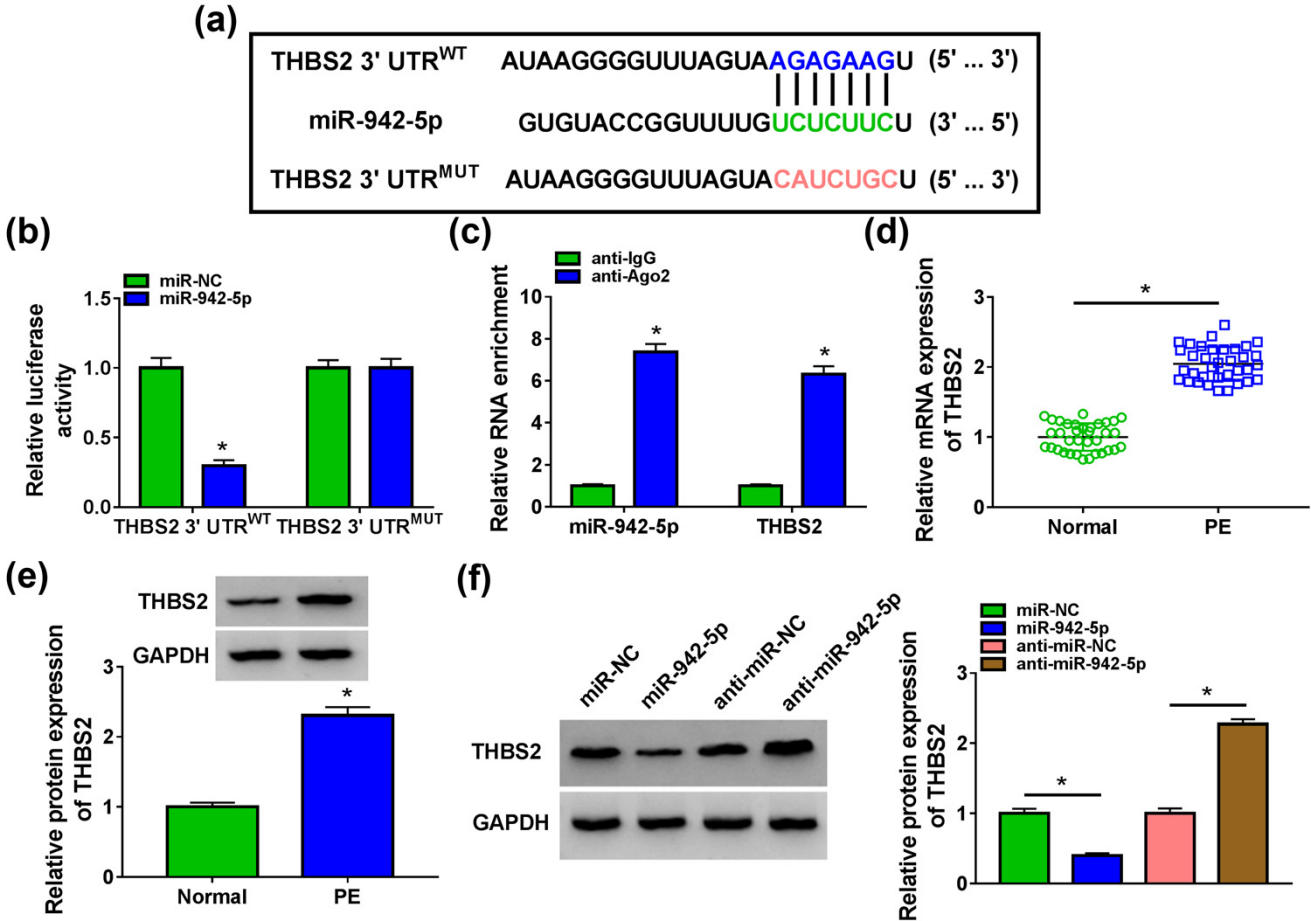
miR-942-5p could target THBS2. (a) The sequences of THBS2 3′-UTR^WT/MUT^ vectors were presented. Dual-luciferase reporter assay (b), and RIP assay (c) were used to verify the interaction between miR-942-5p and THBS2. (d and e) The mRNA and protein expression of THBS2 in the placental tissues of PE patients and normal pregnant women were examined by qRT-PCR and WB analysis. (f) The protein expression of THBS2 was determined by WB analysis in HTR-8/SVneo cells transfected with miR-942-5p mimic and inhibitor. *P < 0.05.

The targetScan software was used to predict the target of miR-942-5p, and the results suggested that miR-942-5p could bind with the 3′-UTR of THBS2 (Figure 5a). The results of dual-luciferase reporter assay indicated that miR-942-5p mimic could inhibit the luciferase activity of THBS2 3′-UTR^WT^ vector, while it had no effect on that of the THBS2 3'-UTR^MUT^ vector (Figure 5b). Also, the enrichments of miR-942-5p and THBS2 were significantly increased in anti-Ago2 (Figure 5c). These results suggested that miR-942-5p could interact with THBS2. Moreover, we discovered that THBS2 was upregulated in the placental tissues of PE patients at the mRNA level and protein level (Figure 5d and e). After overexpressed and silenced miR-942-5p using miR-942-5p mimic and inhibitor, we found that THBS2 protein expression could be reduced by miR-942-5p overexpression and enhanced by miR-942-5p inhibition (Figure 5f). Our data revealed that miR-942-5p had a negative regulation on THBS2 expression.

### 3.6 MiR-942-5p promoted the biological functions of HTR8/SVneo cells by targeting THBS2

**Figure 6:**
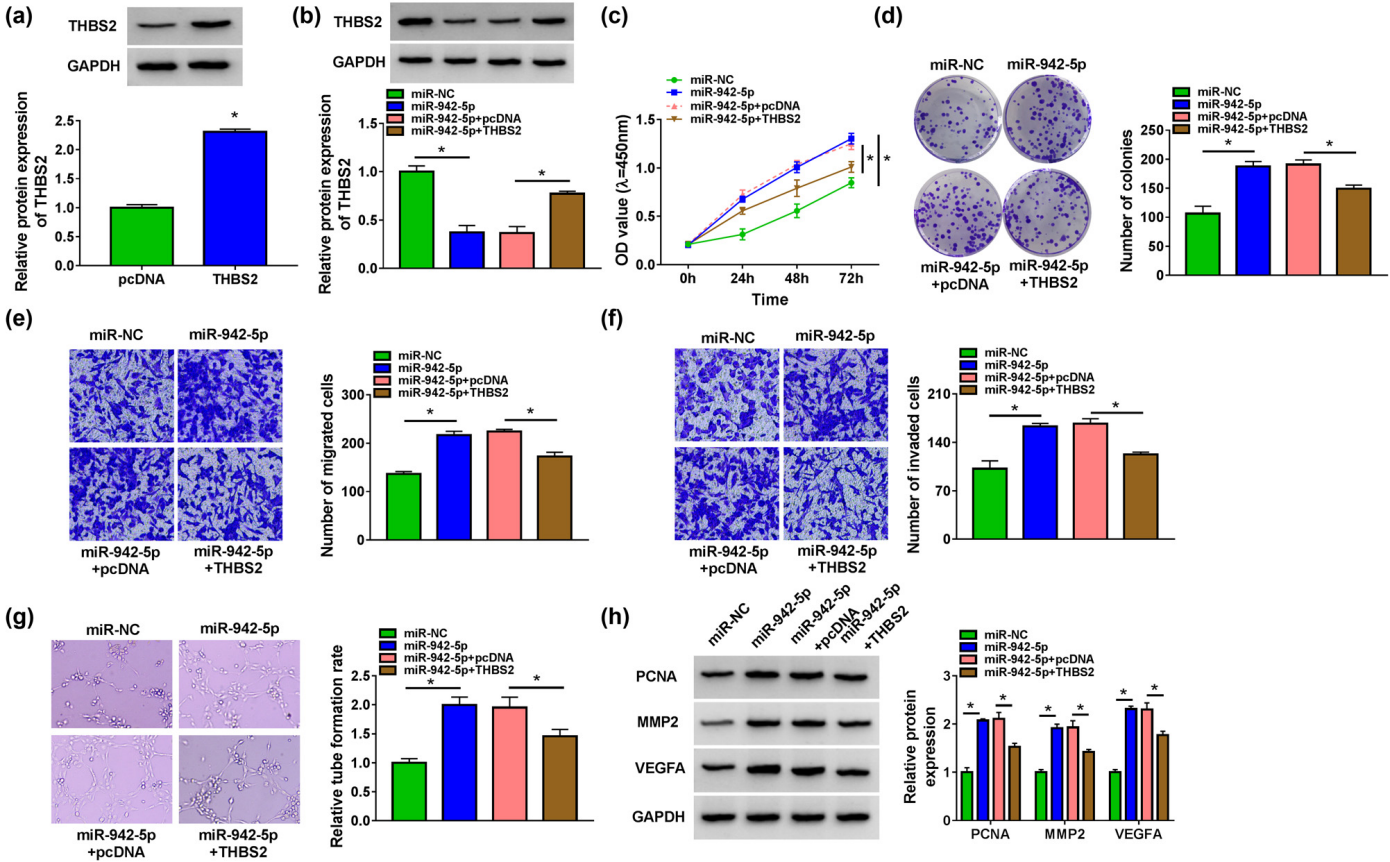
Effects of miR-942-5p and THBS2 on the biological functions of HTR8/SVneo cells. (a) The protein expression of THBS2 was detected by WB analysis to assess the transfection efficiency of THBS2 overexpression vector. (b–h) HTR-8/SVneo cells were transfected with miR-NC, miR-942-5p, miR-942-5p + pcDNA, or miR-942-5p + THBS2. (b) The protein expression of THBS2 was examined by WB analysis. CCK8 assay (c), colony formation assay (d), Transwell assay (e and f), and tube formation assay (g) were measured by cell proliferation, migration, invasion, and angiogenesis, respectively. (h) The protein levels of PCNA, MMP2, and VEGFA were determined by WB analysis. *P < 0.05.

To confirm that miR-942-5p targeted THBS2 to regulate PE progression, the rescue experiments were performed. First, we confirmed that THBS2 overexpression vector could remarkably increase THBS2 protein expression in HTR8/SVneo cells (Figure 6a). Then, HTR8/SVneo cells were co-transfected with miR-942-5p mimic and THBS2 overexpression vector. Through measuring THBS2 protein expression, we discovered that the decreasing effect of miR-942-5p mimic on THBS2 protein expression could be enhanced by THBS2 overexpression vector (Figure 6b). MiR-942-5p could promote the viability, the number of colonies, and the number of migrated and invaded cells, while these effects could be reversed by THBS2 overexpression (Figure 6c–f). Meanwhile, miR-942-5p mimic also enhanced the tube formation rate and increased the protein expression of PCNA, Ki67, MMP9, MMP2, and VEGFA. However, THBS2 overexpression also could abolish these effects (Figure 6g and h and Figure A1c). Hence, our data suggested that miR-942-5p targeted THBS2 to facilitate the proliferation, migration, invasion, and angiogenesis of HTR8/SVneo cells.

### 3.7 circ_0085296 positively regulated THBS2 via sponging miR-942-5p

**Figure 7:**
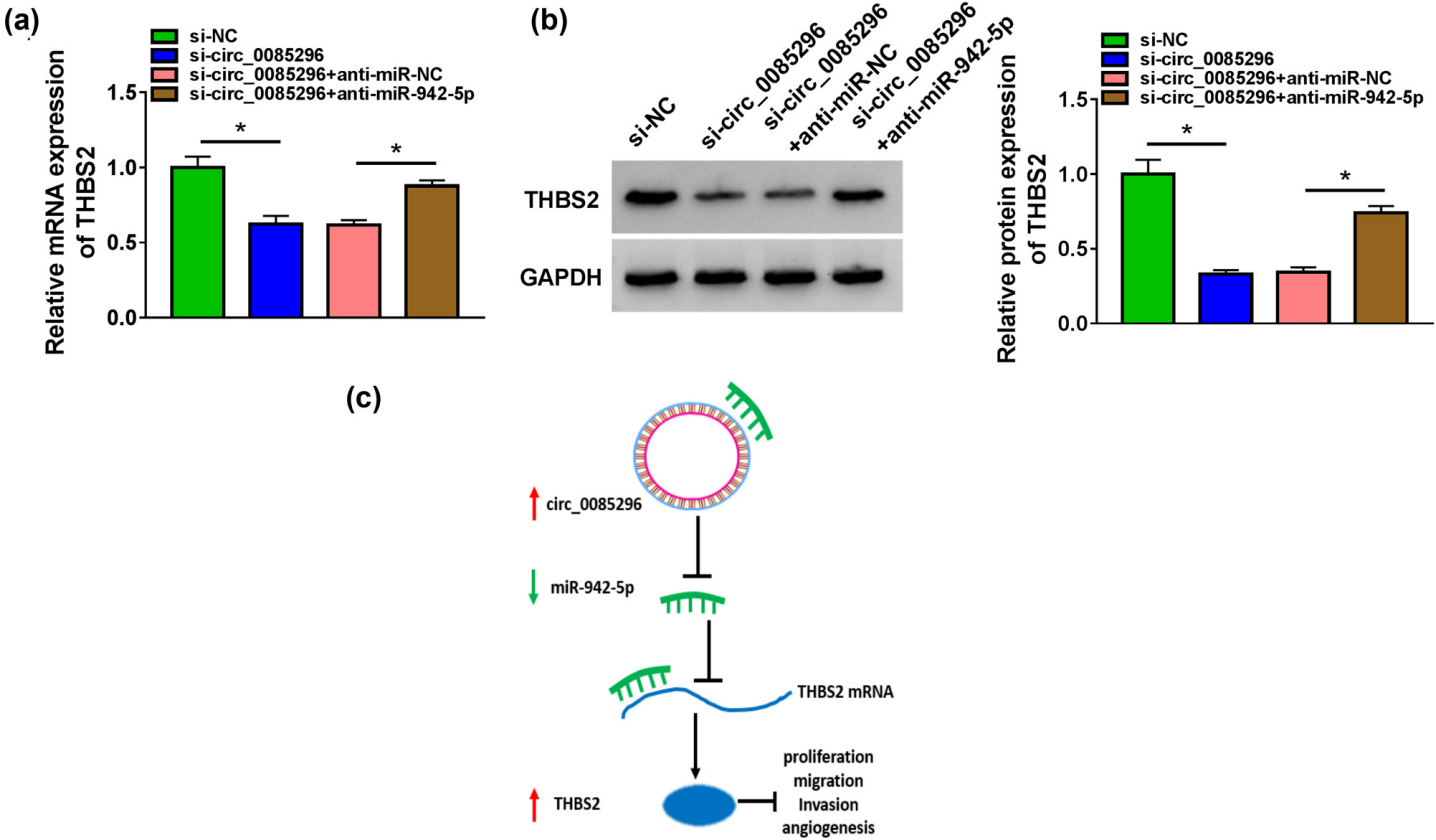
circ_0085296 positively regulated THBS2 via sponging miR-942-5p. (a and b) The mRNA and protein expression of THBS2 was measured by qRT-PCR and WB analysis in HTR-8/SVneo cells co-transfected with si-circ_0085296 and anti-miR-942-5p. (c) The main idea diagram of this study. *P < 0.05.

To further analyze the regulation of circ_0085296 on THBS2 expression, we detected the mRNA and protein expression of THBS2 in HTR8/SVneo cells transfected with si-circ_0085296 and anti-miR-942-5p. As shown in Figure 7a and b, circ_0085296 knockdown markedly reduced the mRNA and protein expression of THBS2, while these effects could be reversed by miR-942-5p inhibitor. Above all, our data revealed that circ_0085296 could sponge miR-942-5p to upregulate THBS2, thereby inhibiting the proliferation, migration, invasion, and angiogenesis of HTR8/SVneo cells (Figure 7c).

## 4 Discussion

At present, the treatment principle for PE is symptomatic treatment such as spasmolysis, sedation, and hypotension, and pregnancy must be terminated when it is serious. Therefore, early treatment and early detection are very important for PE patients. The etiology of PE may involve maternal, placental, and fetal factors, including abnormal invasion of trophoblast cells, abnormal immune regulation, endothelial cell injury, genetic, and nutritional factors [16,17,18,19]. Among them, abnormal invasion of trophoblastic cells may be an important cause of PE. In this research, we assessed the role of circ_0085296 in the function of trophoblast cells. Our data confirmed that circ_0085296 had an inhibition on the proliferation, migration, and invasion of HTR-8/Svneo cells, which was similar to the previous study [15]. In addition, our study also showed that circ_0085296 restrained the angiogenesis of trophoblast cells. Our study once again confirmed the suppressive effect of circ_0085296 on the function of trophoblast cells, suggesting that targeted inhibition of circ_0085296 might be an effective method to alleviate PE progression.

In the terms of mechanism, circ_0085296 was discovered to serve as miR-942-5p sponge. Many studies had shown that miR-942-5p participated in the regulation of cancer malignant progression as a tumor promoter or inhibitor [20,21,22]. Luo et al. reported that miR-942-5p could inhibit the inflammation and apoptosis of LPS-induced kidney cells, showing that it might relieve septic acute kidney injury [23]. In PE-related studies, miR-942-5p was discovered to promote HTR-8/Svneo cells’ proliferation, migration, and invasion [24]. Additionally, lowly expressed miR-942-5p was found to have high diagnostic value in the plasma of PE patients, knockdown of which could reduce trophoblast cell invasion and angiogenesis [25]. In our data, miR-942-5p was downregulated in PE patients. Function experiments revealed that miR-942-5p promoted the proliferation, migration, invasion, and angiogenesis of HTR-8/Svneo cells, and its inhibitor also reversed the regulation of circ_0089396 knockdown on the function of HTR-8/Svneo cells. These data showed the negative role of miR-942-5p in PE progression and confirmed that circ_0085296 sponged miR-942-5p to participate in PE progression.

THBS2, a member of the Thrombocyte reactive protein family, has a binding site for metal matrix transferase to involve in the regulation on cell invasion and migration [26,27]. Many studies had suggested that knockdown of THBS2 could promote cancer cell metastasis, proliferation, and angiogenesis [28,29]. A recent study showed that circ-AK2 upregulated THBS2 by sponging miR-454-3p, thereby inhibiting trophoblast cells proliferation, migration, and invasion [30]. Moreover, THBS2 overexpression was found to have an inhibitory role in HTR-8/SVneo cell growth, migration, and invasion [31]. Here we confirmed that THBS2 was targeted by miR-942-5p, and its expression was positively regulated by circ_0085296. Overexpressed THBS2 reversed the positive regulation of miR-942-5p on the biological functions of trophoblast cells, indicating that THBS2 participated in the regulation of miR-942-5p on PE progression. Therefore, our data showed the conclusion that the circ_0085296/miR-942-5p/THBS2 axis regulated PE progression.

In general, our study showed that high expression of circ_0085296 in PE patients might contribute to the progression of PE. circ_0085296 suppressed trophoblast cells proliferation, migration, invasion, and angiogenesis, which was achieved by regulating miR-942-5p/THBS2 pathway. Our research provides new evidence that circ_0085296 is a potential target for PE therapy.
